# Lily polysaccharides alleviate colitis through the microbiota–N8-acetylspermidine–cGAS–STING signaling axis

**DOI:** 10.3389/fmicb.2025.1686902

**Published:** 2025-11-03

**Authors:** Yuanyu Wu, Xiaoyu Wan, Lu Hou, Haolong Zhang, Jialin Wang, Kun Wu, Junwei Shao, Zheyu Song

**Affiliations:** ^1^Department of Gastrointestinal, Colorectal and Anal Surgery, China-Japan Union Hospital of Jilin University, Changchun, China; ^2^Department of Breast Surgery, The Second Hospital of Jilin University, Changchun, China; ^3^School of Clinical Medicine, Changchun University of Chinese Medicine, Changchun, China

**Keywords:** lily polysaccharides, IBD, gut microbiota, N8-acetylspermidine, cGAS-STING

## Abstract

**Introduction:**

With inflammatory bowel disease (IBD) rising and current therapies limited, novel treatments are needed. Natural products are increasingly recognized as promising options for colitis. This study evaluated the therapeutic effects and mechanisms of lily polysaccharides (LP) in dextran sulfate sodium (DSS)–induced ulcerative colitis (UC).

**Methods:**

LP was administered in a DSS-induced UC model. Gut microbiota composition was profiled by sequencing, and metabolites were assessed with a focus on N8-acetylspermidine (N8AS). In vitro assays examined LP’s impact on N8AS production and intestinal barrier repair. Exogenous N8AS supplementation was tested for anti-colitic effects. Activation of the cyclic GMP–AMP synthase–stimulator of interferon genes (cGAS–STING) pathway and pro-inflammatory cytokine production were evaluated.

**Results:**

LP significantly alleviated colitic symptoms and restored microbial homeostasis, enriching beneficial taxa such as Bacteroides. LP markedly increased N8AS levels; in vitro, LP enhanced N8AS production, and exogenous N8AS supplementation alleviated colitis. Mechanistically, both LP and N8AS inhibited cGAS–STING pathway activation, reduced pro-inflammatory cytokines, and promoted intestinal barrier repair in vitro.

**Discussion:**

LP exerts anti-colitic activity through the microbiota/N8AS/cGAS–STING axis, linking microbial regulation, metabolic modulation, and immune signaling suppression. These findings support LP as a promising natural therapeutic for UC and provide novel insights into the beneficial effects and preliminary mechanisms of N8AS.

## Highlights

- Lily polysaccharides (LP) restore microbial balance in DSS-induced colitis.
- LP upregulates N8-acetylspermidine (N8AS), a protective microbial metabolite.
- LP and N8AS co-inhibit the cGAS–STING pathway and reduce intestinal inflammation.
- LP alleviates colitis via the microbiota/N8AS/cGAS–STING immunometabolic axis.

## 1 Introduction

Inflammatory bowel disease (IBD) is a chronic, relapsing, and nonspecific inflammatory disorder of the gastrointestinal tract, encompassing ulcerative colitis (UC) and Crohn’s disease (CD) (Dowdell and Colgan, 2021). According to the 2019 Global Burden of Disease (GBD) report, an estimated 4.9 million individuals were affected by IBD worldwide, with China and the United States showing the highest prevalence (Danpanichkul et al., 2024; Dharni et al., 2024). In China, IBD has emerged as one of the most prevalent gastrointestinal disorders. Nevertheless, its etiology and pathogenesis remain complex and poorly understood.

Current research has primarily highlighted the complex interplay between IBD and genetic, environmental, immune, and microbial factors (Cannarozzi et al., 2024; Jauregui-Amezaga and Smet, 2024; Yeshi et al., 2024; Zhang C. et al., 2024). Accordingly, current therapeutic strategies still rely predominantly on immunomodulators, aminosalicylates, and corticosteroids, whereas biologic agents, such as anti-TNF-*α* inhibitors, are mainly reserved for the management of moderate-to-severe IBD (Hong and Moon, 2024; Kim and Lee, 2024; Smith et al., 2024). Nevertheless, clinical evidence indicates that approximately 30% of patients exhibit primary non-responsiveness to TNF-α therapy, and with prolonged treatment, nearly 45% experience a significant loss of therapeutic efficacy, representing a major challenge in current clinical practice (Huang et al., 2024; Kim et al., 2024; Lewandowski et al., 2024). Therefore, elucidating the mechanisms underlying the pathogenesis and progression of IBD and identifying novel molecular targets are of critical importance for improving therapeutic outcomes and facilitating drug development.

The etiology and pathogenesis of IBD are characterized by considerable complexity and frequent relapse, and are generally attributed to an imbalance between the gut microbiota and mucosal immunity (Belei et al., 2024; Prins et al., 2024; Wang X. et al., 2024; Zhang Z. et al., 2024). Dysbiosis typically manifests as an enrichment of pathogenic taxa and depletion of commensal species, thereby fostering a pro-inflammatory microenvironment and compromising intestinal barrier integrity (Han et al., 2024; Lao et al., 2024; Li M. et al., 2024). Moreover, bacterial toxins, immune-modulatory proteins, and persistent colonization by pathogenic microbes increase mucosal permeability and impair epithelial barrier function (Gerner et al., 2022; Kong et al., 2024; Ma et al., 2024). Overgrowth of pathogenic or opportunistic bacteria further alters microbial metabolic profiles, thereby provoking inflammation and tissue damage (Lin et al., 2024; Recharla et al., 2023; Zhao et al., 2024). This impaired barrier function and subsequent microbial translocation exacerbate mucosal injury, perpetuating a vicious cycle (Devereaux et al., 2024; Wu et al., 2024). Clinical studies have consistently demonstrated that IBD patients exhibit markedly reduced microbial diversity and ecological stability, characterized by a depletion of *Firmicutes* alongside an expansion of *Bacteroidetes* and facultative anaerobes (Andoh and Nishida, 2023; De Caro et al., 2024; Ilan et al., 2024; Lewis et al., 2024; Rashed et al., 2022; Simpson et al., 2023; Yang et al., 2024). Collectively, these findings underscore that alterations in gut microbiota play a pivotal role in the onset and progression of UC, although the precise mechanisms by which they disrupt the intestinal barrier and promote IBD remain to be fully clarified.

Lily polysaccharides (LP) are heterogeneous polysaccharides extracted from the bulbs of Lilium species, which are recognized for their nutritional value and diverse biological activities (Zhou et al., 2021). Previous studies have demonstrated that LP exert multiple biological functions, including immunomodulatory, anti-tumor, hypoglycemic, and antioxidant effects (Gao et al., 2022; Han and Xie, 2013; Kang et al., 2022). Notably, LP exhibit pronounced immunoregulatory activity, partly through the enhancement of macrophage phagocytosis (Guo et al., 2024), and they are also capable of mitigating oxidative stress and restoring cellular homeostasis (Xu et al., 2016). With respect to gut health, dietary lily intake has been reported to alleviate DSS-induced colitis in mice, although the specific active constituents responsible have not been fully identified (Okazaki et al., 2016). Furthermore, LP have been shown to ameliorate alcoholic fatty liver disease by improving microbial diversity and modulating microbial metabolic pathways, including primary bile acid biosynthesis and amino acid metabolism. Nevertheless, studies specifically addressing the role of LP in intestinal inflammation, particularly in murine models of colitis, remain limited (Li et al., 2025). Therefore, the present study aims to elucidate the therapeutic potential and underlying mechanisms of LP in alleviating ulcerative colitis, with a particular focus on their capacity to regulate gut microbiota composition, microbial metabolites, and inflammation-associated signaling pathways.

## 2 Materials and methods

### 2.1 Reagents

N8-acetylspermidine dihydrochloride was purchased from MCE (USA), and N8-acetylspermidine (N8AS) was purchased from Sigma Chemical (USA). STING (Cat # 19851-1-AP), IRF3 (Cat # 11312-1-AP), p-IRF3 (Cat # 11312-1-AP) were purchased from proteintech (China), TBK1 (CAT # 3504 T). p-TBK1 (CAT # 5483S) was purchased from CST (USA) and cGAS (CAT # ZRB1406) was purchased from Sigma-Aldrich (USA). ELISA kits for the detection of IL-1β, IL-6 and TNF-*α* were purchased from Biolegend (USA).

### 2.2 Preparation and purification of LP

Fresh lily bulbs were thoroughly washed to remove extraneous materials, dried to eliminate residual surface moisture, and homogenized into a fine slurry using a mechanical grinder. The slurry was mixed with distilled water and subjected to ultrasonic treatment to facilitate cell disruption, followed by further homogenization using a cryogenic wall-breaking device. After wall disruption, the slurry was extracted with hot water in a water bath, and the resulting mixture was filtered to remove insoluble residues. The filtrate was concentrated and subjected to ethanol precipitation, after which deproteinization was performed to obtain the crude polysaccharide fraction. For purification, ethanol was added stepwise until the final concentration reached 90%, and the solution was kept at 4 °C to precipitate polysaccharides. The precipitate was then collected by centrifugation and re-dissolved in distilled water or other suitable solvents. The final product obtained was LP with an estimated purity of ~80%.

### 2.3 Construction of animal disease model

Male ICR mice aged 7–8 weeks were used in this study. All mice were acclimatized for 7 days under standard conditions (25 °C, 12 h light/dark cycle, ad libitum access to food and water). A total of 45 mice were randomly divided into three independent experimental sets, each with three groups (*n* = 5 per group):

Experiment 1 (to evaluate the therapeutic effect of lily polysaccharides, LP): Control (blank control), DSS (ulcerative colitis model), DSS + LP (LP-treated group, 150 mg/kg); Experiment 2 (to evaluate the effect of fecal microbiota transplantation): Control, DSS, F-DSS (fecal microbiota transplantation from DSS mice), F-DSS + LP (fecal microbiota transplantation from DSS + LP mice); Experiment 3 (to evaluate the effect of N8AS): Control, DSS, DSS + N8AS (N8AS-treated group, 25 mg/kg).

For the colitis model, mice received 2% DSS in drinking water for 7 consecutive days. During this period, body weight and fecal consistency were monitored daily. LP and N8AS dihydrochloride were administered orally once daily during the 7-day DSS exposure. On day 9, mice were anesthetized with 1% sodium pentobarbital (50 mg/kg, i.p.) and euthanized by cervical dislocation. Colon length was measured, and colon tissues and contents were collected for further analysis. All animal procedures were approved by the Animal Experimental Welfare and Ethics Committee of Jilin University (SY202408003).

### 2.4 Disease activity index

DAI was defined as the sum of weight loss scores (0–4), fecal consistency scores (0–4), and fecal occult blood scores (0–4) (Li et al., 2021).

### 2.5 ELISA

The TNF-*α* kit (Cat# 430907), IL-1β kit (Cat# 432615), and IL-6 kit (Cat# 431315) were all purchased from BioLegend (USA). ELISA assays were performed according to the manufacturer’s instructions. Briefly, pre-coated antibody plates were used, and colon tissue homogenate supernatants and standards were added. After incubation and washing, detection antibodies and enzyme conjugates were applied, followed by color development. The optical density was measured at 450 nm to calculate cytokine concentrations in the samples.

### 2.6 H&E stain

A segment of distal colon (~1 cm from the anus) was collected from each mouse. A 0.5 cm section of the tissue was thoroughly rinsed and fixed in 4% paraformaldehyde for 24 h. After fixation, the tissue was dehydrated, cleared, embedded in paraffin, and sectioned at a thickness of 5–8 μm. Following sectioning, the slides were dried, dewaxed, and rehydrated, then stained with H&E. Histological images were captured using a light microscope. The histology of the colon was evaluated by an optical microscope, and the pathological severity was scored based on inflammatory cell infiltration (0–4), goblet cell depletion or decreased mucus accumulation (0–4), mucosal thickening (0–4), building destruction (0 or 3–4), and crypt loss (0 or 3–4) (Johansson et al., 2014).

### 2.7 Immunofluorescence

Paraffin-embedded mouse colon tissue sections were dried, dewaxed, and rehydrated, followed by permeabilization with 0.2% Triton X-100 for 15 min at room temperature. The sections were then washed with PBST (PBS containing 0.1% Tween-20). After washing, non-specific binding was blocked by incubating the sections in PBST containing 5% donkey serum for 2 h at room temperature. Subsequently, the samples were washed again with PBST and incubated overnight at 4 °C with a primary antibody against MUC2 (Rabbit polyclonal IgG, Proteintech, Catalog No. 27675-1-AP, unconjugated; 1:200 dilution). Following primary antibody incubation, the sections were washed and then incubated with a fluorescent-conjugated secondary antibody at room temperature for 1 h. After thorough washing with PBST, nuclei were counterstained with DAPI-containing anti-fade mounting medium. Fluorescence signals were visualized and captured using a laser scanning confocal microscope.

### 2.8 Gut microbiota sequencing

The colon contents of mice were collected and stored at −80 °C for microbiome analysis. This trial was designed to investigate whether LP could modulate gut microbial composition and metabolites to alleviate DSS-induced colitis. A total of 10 mice (*n* = 5) were included in the experiment. After sample pretreatment, total DNA was extracted using the OMEGA Soil DNA Kit (D5635-02, Omega Bio-Tek, Norcross, GA, United States). The bacterial 16S rRNA V3–V4 region was amplified with specific primers, and the purified amplicons were used for library construction. Sequencing was performed on the Illumina NovaSeq (PE250) or MiSeq (PE300) platform.

The sequencing data were processed using QIIME2 (version 2019.4 or 2022.11, depending on project date) for demultiplexing, primer trimming (cutadapt), denoising and chimera removal (DADA2), and feature table construction. Alpha diversity indices (Chao1, Shannon, Simpson) were calculated, and rarefaction curves were generated. Beta diversity was assessed by UniFrac distance metrics with NMDS and PCA for visualization. Taxonomic assignment was performed against the Greengenes database, and ASVs with relative abundance below 0.001% were removed.

Group differences in microbial community composition were evaluated using PERMANOVA (Adonis). Differential taxa were identified by LEfSe. Co-occurrence networks were constructed with SparCC, and microbial metabolic functions were predicted using PICRUSt2 with the KEGG databases. Unless otherwise stated, a two-sided *p* < 0.05 was considered statistically significant.

### 2.9 Fecal bacteria transplantation

To further determine whether the gut microbiota mediates the therapeutic effect of LP in colitis, a fecal microbiota transplantation experiment was conducted. Twenty recipient mice were randomly assigned into four groups (*n* = 5): Control (blank control), DSS (model group), F-DSS (fecal microbiota from DSS mice), and F-DSS + LP (fecal microbiota from DSS + LP-treated mice). Donor feces were collected from mice in the DSS and DSS + LP groups (5 mice per group, not included in the recipient animal count).

To construct a bacterial clearance model, recipient mice were given an antibiotic cocktail in their drinking water for 28 days, consisting of penicillin (1 g/L), metronidazole (1 g/L), neomycin (1 g/L), and vancomycin (0.5 g/L). After antibiotic treatment, acute colitis was induced with 2% DSS administered for 7 days. Donor fecal samples were homogenized in sterile PBS (100 mg/mL) and centrifuged at 2000 rpm for 3 min. The resulting supernatant was orally administered to recipient mice once daily for 7 consecutive days.

### 2.10 Non-targeted metabolite sequencing

Metabolomic analysis was performed using the Perseno Gene Cloud.^1^ The colonic contents of mice were collected, immediately frozen, and stored at −80 °C. Metabolites were extracted from the samples by organic solvent protein precipitation, followed by non-targeted LC–MS analysis on a Thermo Vanquish UHPLC system coupled with an Orbitrap Exploris 120 mass spectrometer. Chromatographic separation was carried out with an ACQUITY UPLC HSS T3 column under both positive and negative ion modes, and high-resolution MS/MS data were acquired. The raw data were processed with Compound Discoverer™ 3.3 for peak extraction, alignment, normalization, and metabolite annotation against multiple databases (HMDB, KEGG, LIPID MAPS, mzCloud, MoNA, and NIST). Univariate and multivariate statistical analyses were then conducted to identify differential metabolites between groups.

### 2.11 Cell culture and treatment

The Caco-2 and RAW264.7 cells were transferred into the medium (90% DMEM high glucose medium, 10% fetal bovine serum) and cultured in a constant temperature incubator at 37 °C and 5% CO_2_. The medium was changed every day, and the cells were passaged when the cell density was 80–90%, 2–3 times a week. The concentration of LP was 200 μg / ml, and the concentration of N8-acetylspermidine dihydrochloride was 10 μM. After 24 h of treatment, the cells were collected for subsequent experiments.

### 2.12 qRT-PCR

The total RNA of mouse colon tissue was extracted, and the mRNA expression of key genes of tight junction in mouse colon tissue was determined by qRT-PCR. The primer sequences are shown in Table 1.

**Table 1:** The primer sequences.

Gene	Sequences (5′-3′)	GenBank accession number
*ZO-1*	(F) TGCCATTACACGGTCCTCTG (R) GGTTCTGCCTCATCATTTCCTC	NM_003257
*Occludin*	(F) CCCATCTGACTATGTGGAAAGA (R) AAAACCGCTTGTCATTCACTTTG	NM_002538
*MUC2*	(F) TGCCTGGCCCTGTCTTTG (R) CAGCTCCAGCATGAGTGC	NM_002457.5
*β-actin*	(F) TCATGAAGTGTGACGTGGACATC (R) TGCATCCTGTCGGCAATG	NM_001101

### 2.13 Western blot

Colon tissues or Caco-2 cells were collected and lysed using NP-40 protein lysis buffer supplemented with 3–5 stainless steel beads. After homogenization, samples were incubated on ice for 30 min to ensure complete lysis. Lysates were then centrifuged at 12,000 rpm for 10 min at 4 °C, and the resulting supernatants were collected as total protein extracts. Protein concentrations were quantified using a BCA protein assay kit, and all samples were normalized to the same protein concentration. A total of 40 μg of protein was loaded per well for electrophoresis. SDS-PAGE was performed under the following conditions: 50 V for 30 min, 80 V for 30 min, and 120 V for 30 min. Following electrophoresis, the gel was transferred to a PVDF membrane. The membrane was pre-activated by immersion in methanol for 30 s, then layered on the gel along with transfer filter papers and foam pads. After assembling the transfer cassette, protein transfer was performed at a constant current of 300 mA. Upon completion, the PVDF membrane was carefully removed and blocked with 5% non-fat milk in TBST at room temperature for 1–2 h. The membrane was then washed with TBST and incubated overnight at 4 °C with the primary antibody. After washing, the membrane was incubated with the appropriate secondary antibody at room temperature for 1 h, followed by additional TBST washes. Excess buffer was removed using filter paper, and the membrane was transferred to a chemiluminescence imaging system. An enhanced chemiluminescence (ECL) substrate was evenly applied, and signal detection was performed. Protein band intensity was analyzed using ImageJ software for grayscale quantification.

### 2.14 Statistical analysis

All statistical analyses were conducted using appropriate tools based on the type of dataset.

Microbiome data: Raw sequencing data were processed using QIIME2 (version 2019.4 or 2022.11, depending on project date). After demultiplexing, primer trimming (cutadapt), and denoising (DADA2), amplicon sequence variants (ASVs) were generated. Alpha diversity indices (Chao1, Shannon, Simpson, Faith’s PD, Pielou’s evenness, and Good’s coverage) were calculated in QIIME2, and rarefaction curves were plotted. Beta diversity was assessed based on UniFrac distances and visualized using PCoA, NMDS, and PCA. Taxonomic classification was performed using the Greengenes database, and ASVs with a relative abundance below 0.001% of the total reads were filtered out. Community structure differences between groups were evaluated using PERMANOVA (Adonis function in the R package vegan). Differential taxa were identified via LEfSe. Microbial co-occurrence networks were constructed using SparCC, and functional prediction was performed using PICRUSt2 based on MetaCyc and KEGG databases. Unless otherwise stated, a *p*-value < 0.05 was considered statistically significant.

Metabolomics data: Raw LC–MS data were processed using Compound Discoverer™ 3.3 (version 3.3.2.31, Thermo, Waltham, United States) for peak detection, alignment, total peak area normalization, and metabolite annotation. Reference databases included HMDB, KEGG, LIPID MAPS, mzCloud, MoNA, and NIST_2020_MSMS. Peaks not detected in more than 50% of QC samples were excluded, and missing values were imputed using the Fill Gaps algorithm. The relative standard deviation (RSD) of QC samples was used to assess data reproducibility. Statistical analyses included univariate tests (*t*-test or one-way ANOVA, depending on group number) and multivariate analyses (PCA, PLS-DA, OPLS-DA). Differential metabolites were visualized using volcano plots and hierarchical clustering. False discovery rate (FDR) correction was applied for multiple testing. Significant differential metabolites were defined as those with *p* < 0.05 and |log2 fold change| > 1.

General statistics: GraphPad Prism 8 (GraphPad Software, United States) and Microsoft Excel were used for general statistical analysis and graphing. Differences between two groups were analyzed using unpaired Student’s *t*-tests, and comparisons among multiple groups were performed using one-way ANOVA. A p-value < 0.05 was considered statistically significant. All data are presented as mean ± standard error of the mean (SEM).

## 3 Results

### 3.1 LP inhibited the expression of pro-inflammatory mediators and alleviate colitis

**Figure 1:**
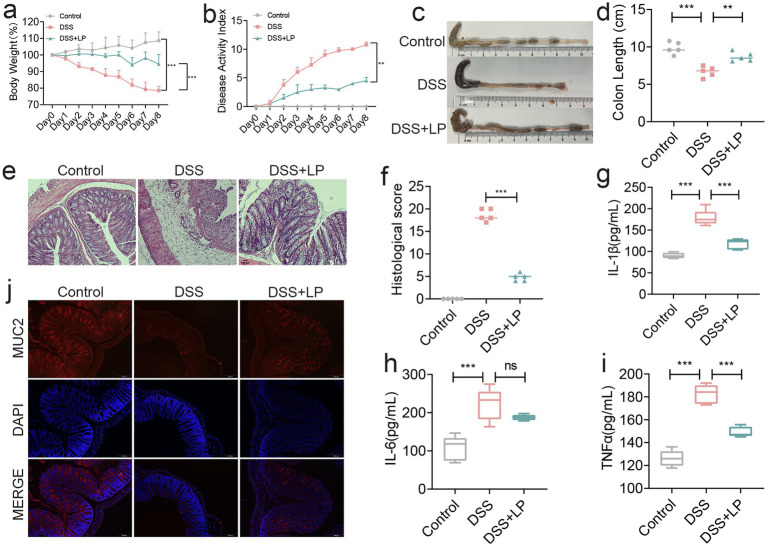
The therapeutic effect of lily polysaccharide on colitis. **(a)** Changes of mice weight. (Day x/Day 0 *100%, *n* = 5). **(b)** Disease activity index. (*n* = 4). **(c,d)** Quantified lengths of colonic tissues isolated from mice after treatment (*n* = 5). **(e)** H&E-stained histological sections of colons. Representative digital photos. **(f)** The pathology score of colons from mice in the different treatment group (*n* = 5). **(g–i)** The levels of pro-inflammatory cytokines IL-1*β*, IL-6 and TNF-*α* in colon of mice were detected by ELISA (*n* = 5). **(j)** The expression levels of MUC2 were determined by immunofluorescence staining in the mouse colon. Representative digital photos. Data are presented as the mean ± SEM. Statistically significant differences are indicated; *p < 0.05, **p < 0.01, *** *p* < 0.001.

To investigate the therapeutic efficacy of LP in colitis, we first assessed their effects in a DSS-induced acute colitis mouse model. As expected, mice in the DSS group exhibited significant body weight loss, and their histopathological scores exceeded 10 by day 8, confirming the successful induction of colitis (Figures 1a,b). LP supplementation markedly attenuated body weight loss and reduced disease activity index (DAI) scores (Figures 1a,b). Moreover, LP treatment significantly restored colon length relative to the DSS group (Figures 1c,d), indicating protective effects against DSS-induced colitis. Histological analysis with H&E staining revealed marked histopathological alterations in the DSS group, including severe inflammatory cell infiltration, goblet cell depletion, and disruption of mucosal architecture. These pathological changes were substantially alleviated by LP treatment (Figures 1e,f). To further evaluate the anti-inflammatory effects of LP, colonic levels of pro-inflammatory cytokines IL-1β, IL-6, and TNF-*α* were quantified. LP administration significantly suppressed DSS-induced upregulation of these cytokines (Figures 1g–i), further supporting its potent anti-inflammatory activity *in vivo*. In addition to inflammation, disruption of the epithelial barrier is a hallmark feature of ulcerative colitis. We therefore examined the expression of MUC2, a key mucin component of the intestinal barrier. DSS treatment led to pronounced downregulation of MUC2 expression, whereas LP supplementation markedly restored its expression, indicating enhanced barrier integrity (Figure 1j). Collectively, these findings demonstrate that LP confers therapeutic benefits in DSS-induced ulcerative colitis by suppressing inflammation and promoting epithelial barrier repair.

### 3.2 LP improved the gut microbiota in colitis

**Figure 2:**
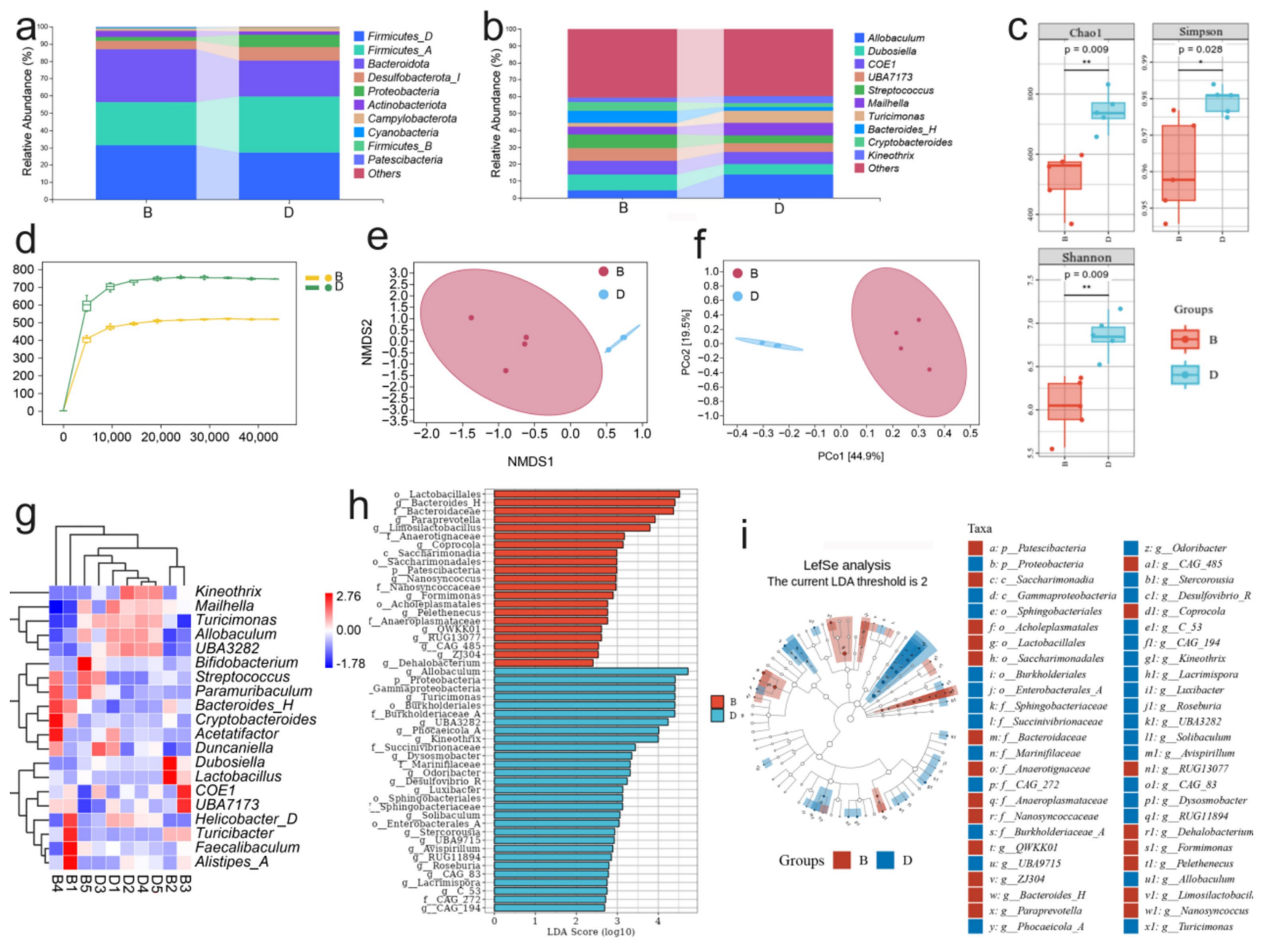
Effect of lily polysaccharide on gut microbiota. “B” represents DSS + LP group, “D” represents DSS group. **(a)** Colonic microbiota composition of mice at the phylum level in different treatment groups. **(b)** Colonic microbiota composition of mice at the genus level in different treatment groups. **(c)** Alpha diversity boxplot of the colonic microbiota in different treatment groups. **(d)** arefaction Curve of the colonic microbiota in different treatment groups. **(e,f)** NMDS analysis of beta diversity among each group (based on Bray–Curtis metric distances). **(g)** Heat map of differential species composition. **(h)** LDA value distribution histogram of significantly different species. **(i)** LEfSe analysis showed the evolutionary branch diagram. Statistically significant differences are indicated; *p < 0.05, **p < 0.01, *** *p* < 0.001.

As the colon harbors the highest density of intestinal microorganisms, it is highly susceptible to microbiota-associated dysregulation during colitis. To evaluate whether LP modulates gut microbial composition, we analyzed the gut microbiota of DSS-induced colitis mice after LP treatment. At the phylum level, LP administration markedly increased the relative abundance of *Bacteroidota* (Figure 2a). At the genus level, significant increases were observed in *Bacteroides-H* and *Dubosiella* (Figure 2b). Diversity analysis revealed significant intergroup differences in Chao1, Shannon, and Simpson indices, indicating pronounced alterations in microbial richness and evenness (Figure 2c). Moreover, rarefaction curves indicated reduced α-diversity following LP treatment (Figure 2d), suggesting that LP reshaped overall microbial diversity. Principal coordinate analysis (PCoA) further demonstrated distinct separation of microbial communities between the LP and DSS groups, with no overlap, highlighting a substantial shift in microbial structure after LP intervention (Figures 2e,f). Compositional analysis revealed that the abundances of *Allobaculum, Turicimonas,* and *UBA3282* were significantly reduced in the LP group, whereas *Turicibacter* and *Bacteroides-H* were significantly enriched compared to the DSS group (Figure 2g). These differential taxa were consistent with the potential microbial biomarkers identified by linear discriminant analysis effect size (LEfSe) (Figures 2h,i). Collectively, these findings indicate that LP profoundly reshapes the gut microbiota during colitis, enriching beneficial bacteria while reducing potentially harmful taxa, thereby contributing to its protective effects against colitis.

**Figure 3:**
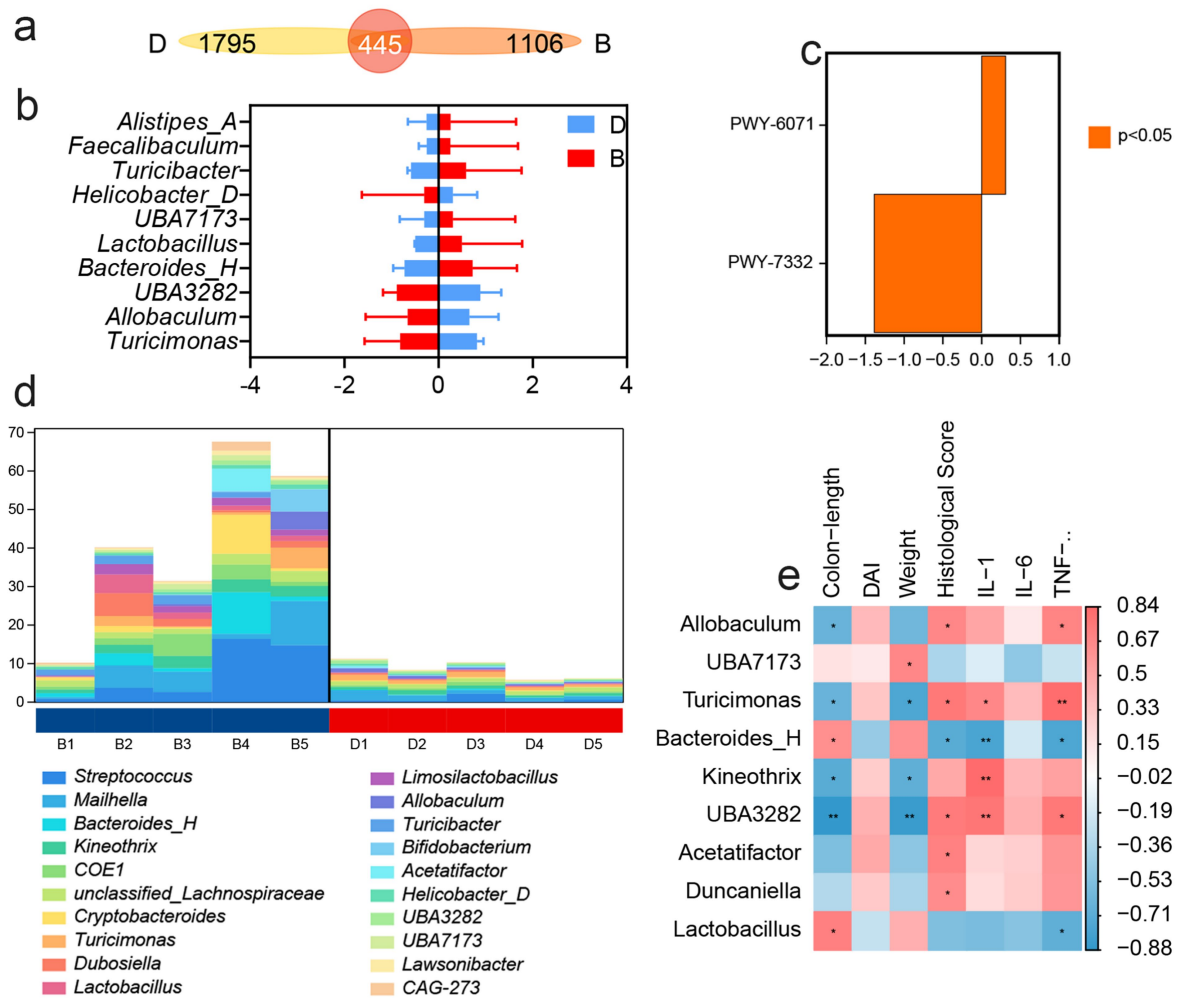
16S rDNA high throughput sequencing. “B” represents DSS + LP group, “D” represents DSS group. **(a)** Venn Graph of significant difference OTUs in the two groups. **(b)** Differential species abundance histogram. **(c)** Analysis of metabolic pathway differences. **(d)** Species composition of metabolic pathways. **(e)** Spearman rank correlation analysis illustrating the relationships among gut microbiota, inflammatory markers and DAI score. Positive correlations are depicted in red, whereas negative correlations are shown in blue. Significance levels are indicated as follows: *p < 0.05, **p < 0.01.

A detailed analysis of gut microbial composition revealed that LP treatment did not markedly alter the overall structure of the core intestinal flora, but it significantly increased the relative abundance of *Bacteroidota* (Supplementary Figure S1). Venn diagram analysis showed that only 445 taxa were shared across groups, whereas the LP group harbored 1,105 unique taxa with differential abundance (Figure 3a). Compared with the DSS group, LP administration increased the relative abundance of *Alistipes-A, Faecalibaculum, Turicibacter, UBA7173, Bacteroides-H*, and *Lactobacillus*, while *Helicobacter-D, Turicimonas, Allobaculum,* and *UBA3282* were significantly decreased (Figure 3b). To further investigate the functional implications of these microbial shifts, predictive functional profiling was conducted. The results showed that differentially abundant taxa were predominantly enriched in biosynthetic metabolic pathways (Supplementary Figure S2), with the most significantly enriched pathway being the superpathway of UDP-N-acetylglucosamine-derived O-antigen building blocks biosynthesis (Figure 3c). The major bacterial contributors to this pathway were *Streptococcus, Mailhella, and Bacteroides-H* (Figure 3d). Correlation analysis between microbial taxa and inflammatory markers demonstrated that *Turicimonas* and *UBA3282* showed positive correlations with disease severity, including histopathological scores and levels of the pro-inflammatory cytokines IL-1β, IL-6, and TNF-*α*, whereas *Bacteroides-H* was negatively correlated with these parameters (Figure 3e). Importantly, LP treatment significantly reduced the abundance of *Turicimonas* and *UBA3282* while enriching *Bacteroides-H,* suggesting that LP beneficially modulates the gut microbiota by selectively shaping key taxa associated with inflammation.

### 3.3 Fecal microbiota transplantation can alleviate colitis by improving gut microbiota

**Figure 4:**
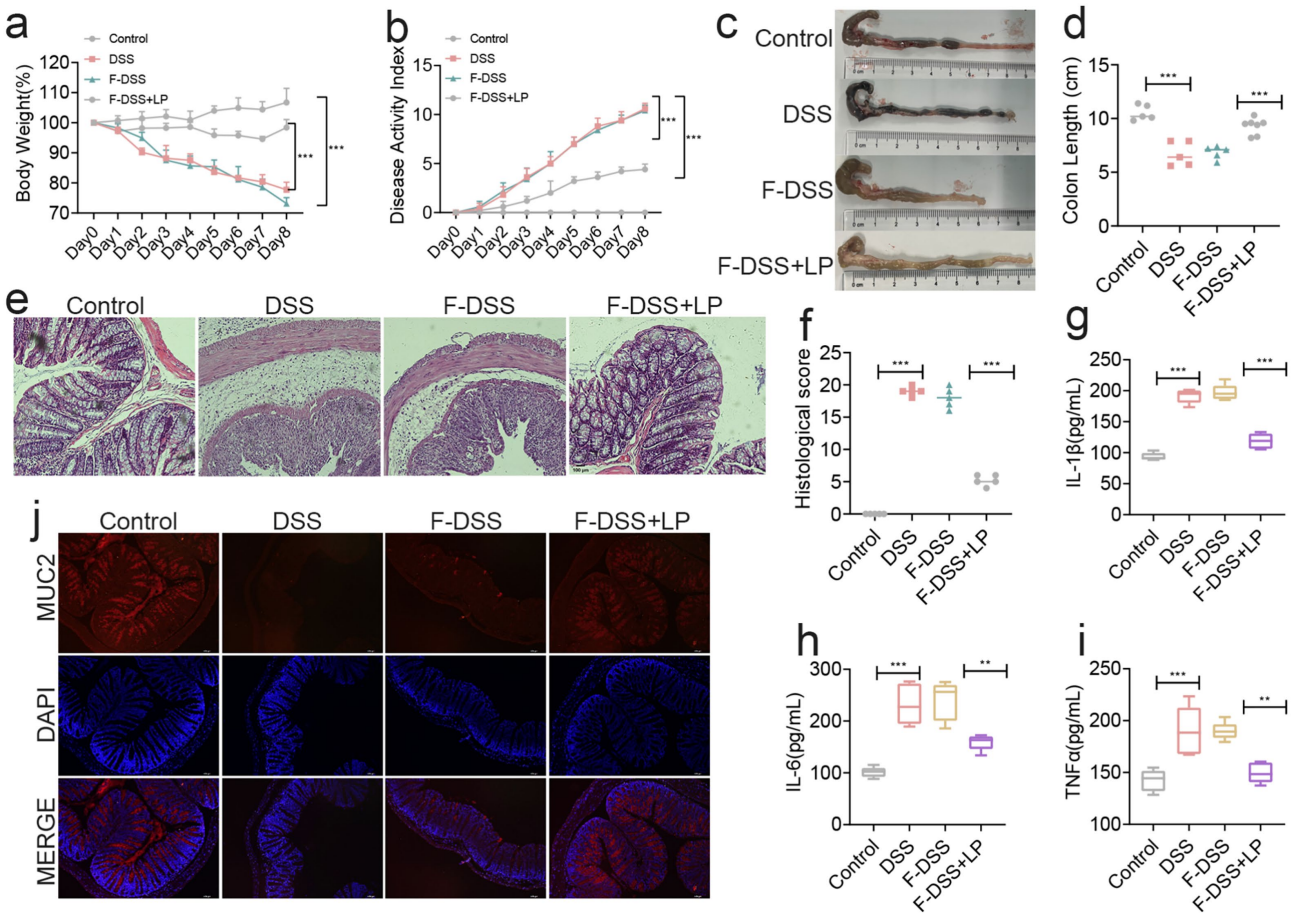
Fecal bacteria transplantation experiments showed that LP could positively regulate the gut microbiota. **(a)** Changes of mice weight. (Day x/Day 0 *100%, *n* = 5). **(b)** Disease activity index (*n* = 5). **(c,d)** Quantified lengths of colonic tissues isolated from mice after treatment (*n* = 5). **(e)** H&E-stained histological sections of colons. Representative digital photos. **(f)** The pathology score of colons from mice in the different treatment group (*n* = 5). **(g–i)** The levels of pro-inflammatory cytokines IL-1β, IL-6 and TNF-α in colon of mice were detected by ELISA (*n* = 5). **(j)** The expression levels of MUC2 were determined by immunofluorescence staining in the mouse colon. Representative digital photos. Data are presented as the mean ± SEM. Statistically significant differences are indicated; *p < 0.05, **p < 0.01, *** *p* < 0.001.

To further validate the modulatory effect of LP on the gut microbiota, we performed fecal microbiota transplantation (FMT) using microbiota derived from LP-treated mice. Assessment of body weight, DAI, and colon length revealed that transplantation of microbiota from the DSS + LP group significantly alleviated DSS-induced colitis symptoms in recipient mice (Figures 4a–d). Histological analysis by H&E staining showed that the F-DSS group exhibited severe histopathological alterations, including marked inflammatory cell infiltration, goblet cell depletion, and disruption of mucosal architecture, which were markedly alleviated in the F-DSS + LP group (Figures 4e,f). In parallel, colonic expression of the pro-inflammatory cytokines IL-1*β*, IL-6, and TNF-α was significantly reduced in the F-DSS + LP group compared with the F-DSS group (Figures 4g–i). Consistently, expression of MUC2, a key marker of intestinal barrier integrity, was significantly upregulated following transplantation of LP-modulated microbiota (Figure 4j). Collectively, these findings indicate that the gut microbiota reshaped by LP plays a pivotal role in ameliorating colitis and that its therapeutic benefits can be transmitted through fecal microbiota transplantation.

### 3.4 LP alleviates colitis by regulating metabolites

**Figure 5:**
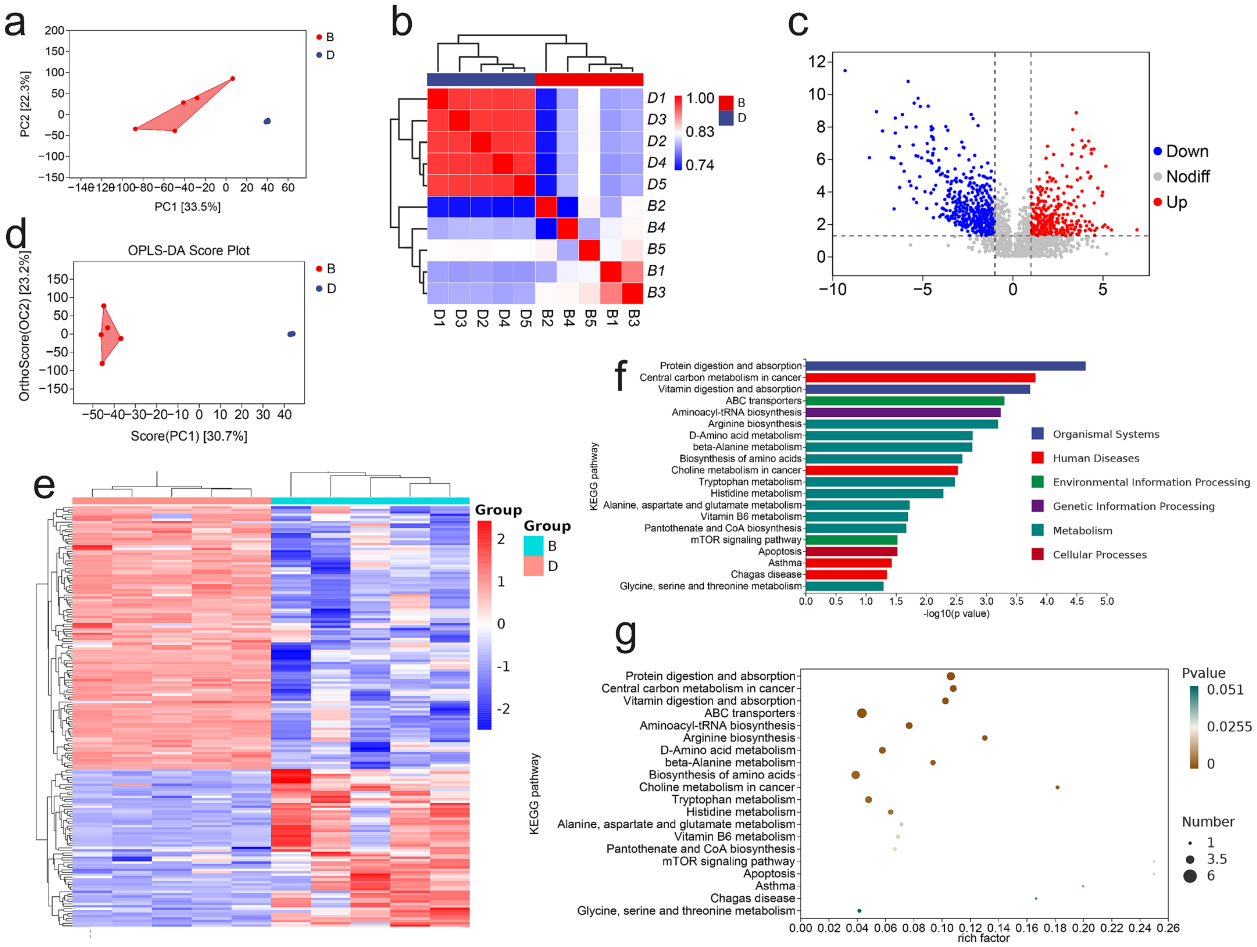
Non-Targeted Metabolomic Analysis of Mouse Gut Microbiota. “B” represents DSS + LP group, “D” represents DSS group. **(a)** PCA of total metabolites. **(b)** Inter-sample correlation assessment. **(c)** Volcano plot of differential metabolites. **(d)** PCA analysis of differential metabolites. **(e)** Clustering analysis of differential metabolites. **(f,g)** KEGG enrichment plot of differential metabolites.

Because gut microbial dysbiosis profoundly affects host metabolic profiles, we employed non-targeted metabolomics to evaluate the impact of LP on fecal metabolites in DSS-induced colitis mice. Comprehensive metabolite profiling revealed distinct differences between the DSS and LP groups, with each group forming clearly separated metabolic subclusters (Figure 5a). Correlation analysis confirmed high within-group consistency and clear between-group differences, underscoring the robustness of LP-induced metabolic shifts (Figure 5b). In total, 650 differential metabolites were identified, including 65 significantly upregulated and 108 downregulated metabolites in the LP-treated group compared with DSS controls (Figure 5c). Hierarchical clustering heatmaps and principal component analysis (PCA) further demonstrated marked differences in metabolic profiles between groups (Figures 5d,e). KEGG pathway enrichment analysis of the annotated differential metabolites revealed significant enrichment in several key metabolic pathways, including arginine biosynthesis, D-amino acid metabolism, β-alanine metabolism, tryptophan metabolism, histidine metabolism, alanine, aspartate and glutamate metabolism, vitamin B6 metabolism, and pantothenate and Coenzyme A biosynthesis (Figures 5f,g). Collectively, these findings indicate that LP significantly modulates the gut metabolome, thereby contributing to its therapeutic effects through regulation of multiple amino acid- and cofactor-related metabolic pathways.

**Figure 6:**
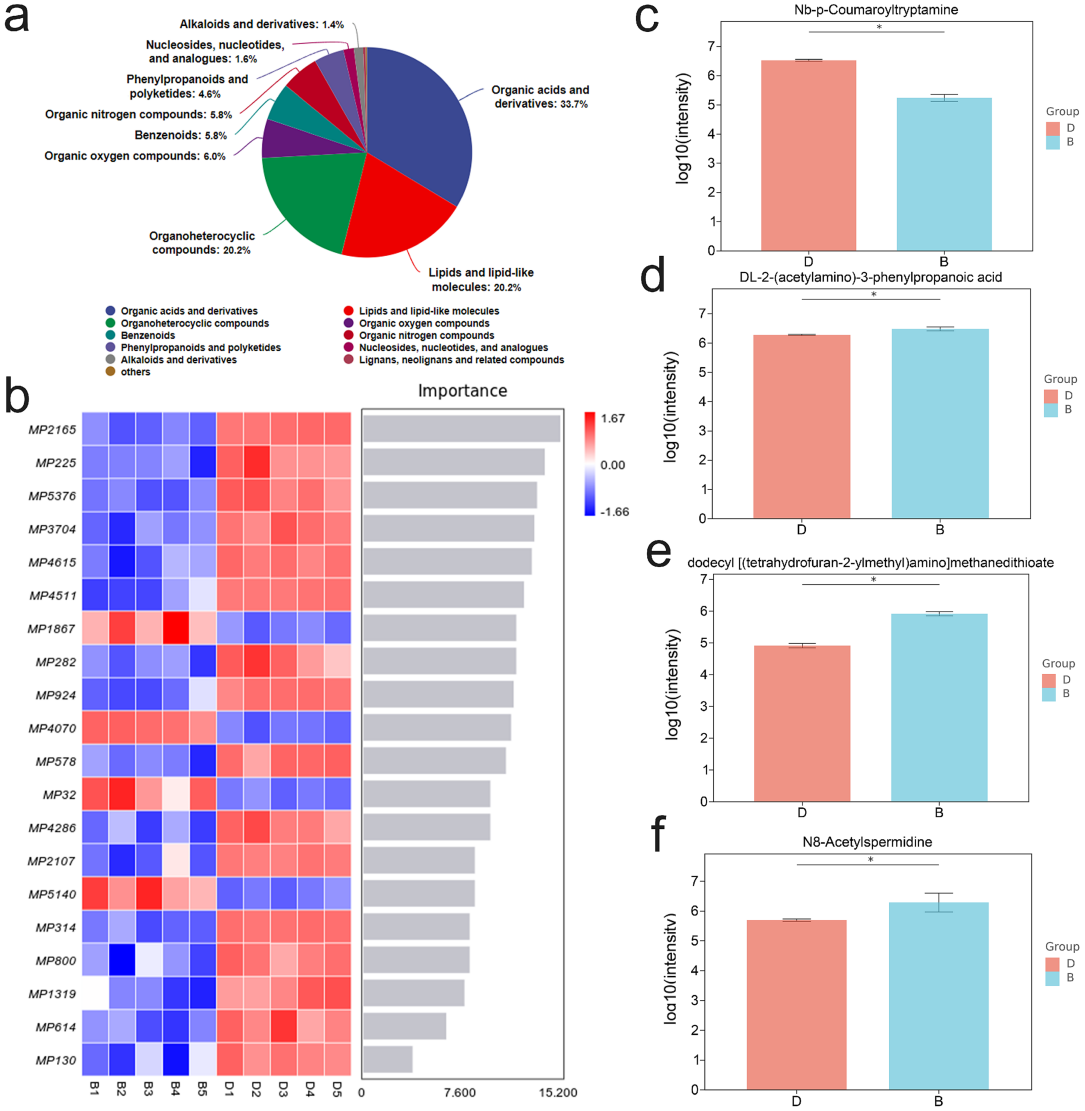
Effect of lily polysaccharide on metabolites. “**b**” represents DSS + LP group, “**d**” represents DSS group. **(a)** Metabolite identification analysis. **(b)** Microbial importance ranking diagram by random forest algorithm. c-f. Column diagram of differential metabolite expression **(c)**, Nb-p-Coumaroyltryptamine; **(d)**, {DL-2-(acetylamino)-3-phenylpropanoic acid; **(e)** dodecyl [(tetrahydrofuran-2-ylmethyl)amino] methanedithioate; **(f)**, N8AS}.

Metabolite profiling revealed that the three most abundant classes of metabolites were organic acids and their derivatives, organic heterocyclic compounds, and lipids and lipid-like molecules (Figure 6a). Further screening identified 20 differential metabolites with the most pronounced changes and potential biological relevance (Figure 6b). Among these, DL-2-(acetylamino)-3-phenylpropanoic acid, dodecyl [(tetrahydrofuran-2-ylmethyl)amino] methanedithioate, and N8-acetylspermidine (N8AS) were significantly increased following LP treatment, whereas Nb-p-Coumaroyltryptamine was significantly decreased (Figures 6c–f). Notably, N8AS is known to play an important role in immune regulation and in promoting longevity.

### 3.5 Replenishing N8AS can effectively alleviate colitis

**Figure 7:**
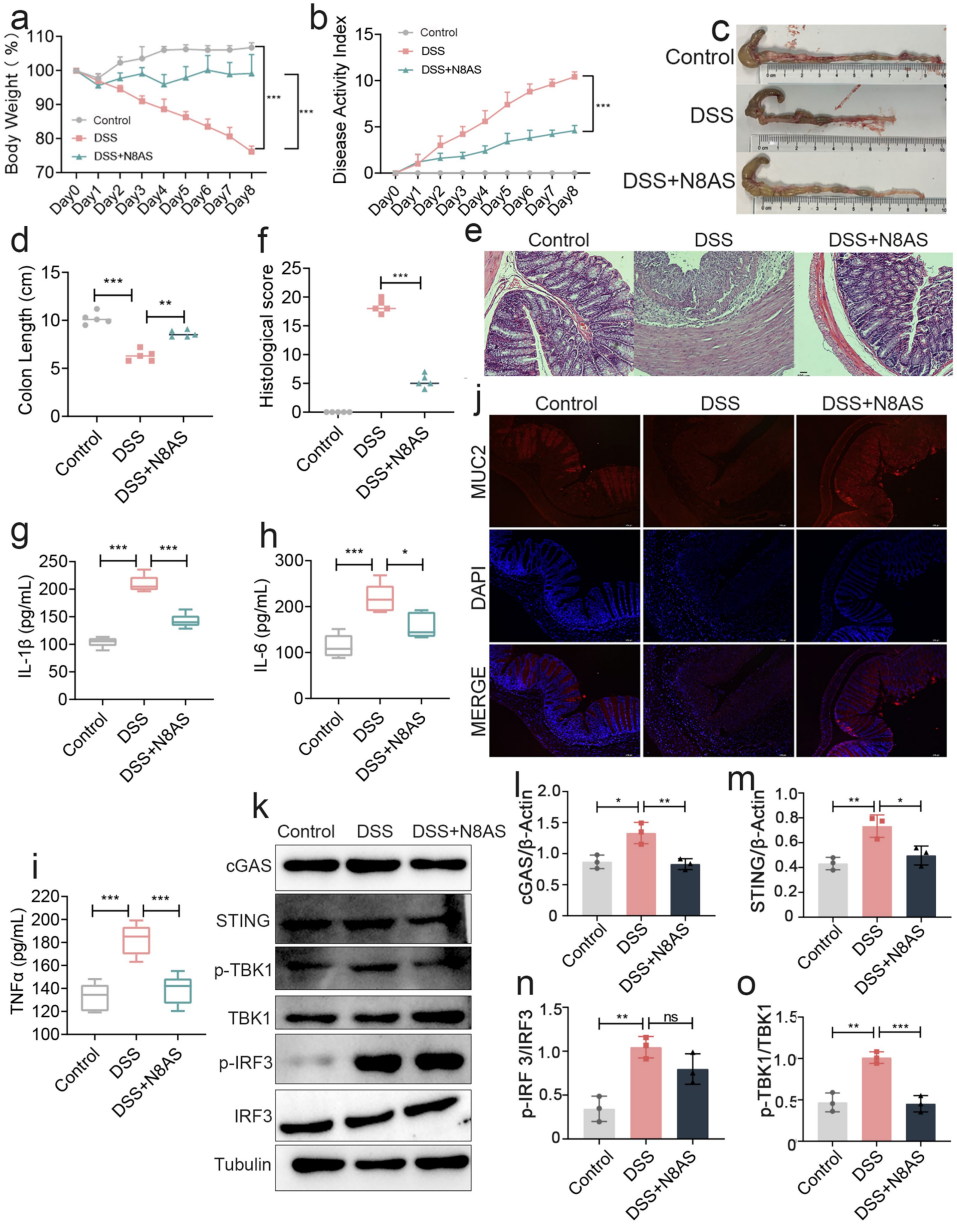
Replenishing N8AS can effectively alleviate enteritis. **(a)** Changes of mice weight.(Day x/Day 0 *100%, *n* = 5). **(b)** Disease activity index. (*n* = 5). **(c,d)** Quantified lengths of colonic tissues isolated from mice after treatment (*n* = 5). **(e)** H&E-stained histological sections of colons. Representative digital photos. **(f)** The pathology score of colons from mice in the different treatment group (*n* = 5). **(g–i)** The levels of pro-inflammatory cytokines IL-1β, IL-6 and TNF-α in colon of mice were detected by ELISA (*n* = 5). **(j)** The expression levels of MUC2 were determined by immunofluorescence staining in the mouse colon. Representative digital photos. **(k–o)** The protein expression levels of cGAS, STING, p-TBK, p-IRF3 were assessed by western blot analysis. Data are presented as the mean ± SEM. Statistically significant differences are indicated; *p < 0.05, **p < 0.01, *** *p* < 0.001.

In the preceding experiments, we observed that LP supplementation significantly increased the levels of N8AS. To further examine the functional role of N8AS, we administered exogenous N8AS dihydrochloride in a DSS-induced colitis mouse model. N8AS treatment significantly attenuated body weight loss and reduced the DAI compared with DSS controls (Figures 7a,b). In addition, colon length was significantly maintained in the N8AS-treated group, indicating alleviation of colonic inflammation (Figures 7c,d). Histological analysis with H&E staining showed that N8AS administration substantially alleviated DSS-induced histopathological injury (Figures 7e,f) and significantly reduced colonic levels of pro-inflammatory cytokines, including IL-1β, IL-6, and TNF-*α* (Figures 7g–i). Immunofluorescence analysis further demonstrated that N8AS effectively restored intestinal epithelial barrier integrity disrupted by DSS treatment (Figure 7j). To explore the underlying mechanisms, we assessed key proteins in the cGAS–STING signaling pathway, including cGAS, STING, phosphorylated TBK1 (p-TBK1), and phosphorylated IRF3 (p-IRF3). DSS exposure strongly activated the cGAS–STING pathway, whereas N8AS supplementation markedly suppressed this activation (Figures 7k–o), suggesting that the anti-inflammatory effects of N8AS are mediated, at least in part, through inhibition of the cGAS–STING signaling cascade. In conclusion, exogenous N8AS supplementation effectively ameliorates DSS-induced colitis, likely through suppression of the cGAS–STING pathway. Nevertheless, further studies are required to elucidate the precise molecular mechanisms by which LP upregulates N8AS to exert its anti-colitic effects.

### 3.6 LP and N8AS inhibit the cGAS-STING pathway and play an anti-inflammatory role

**Figure 8:**
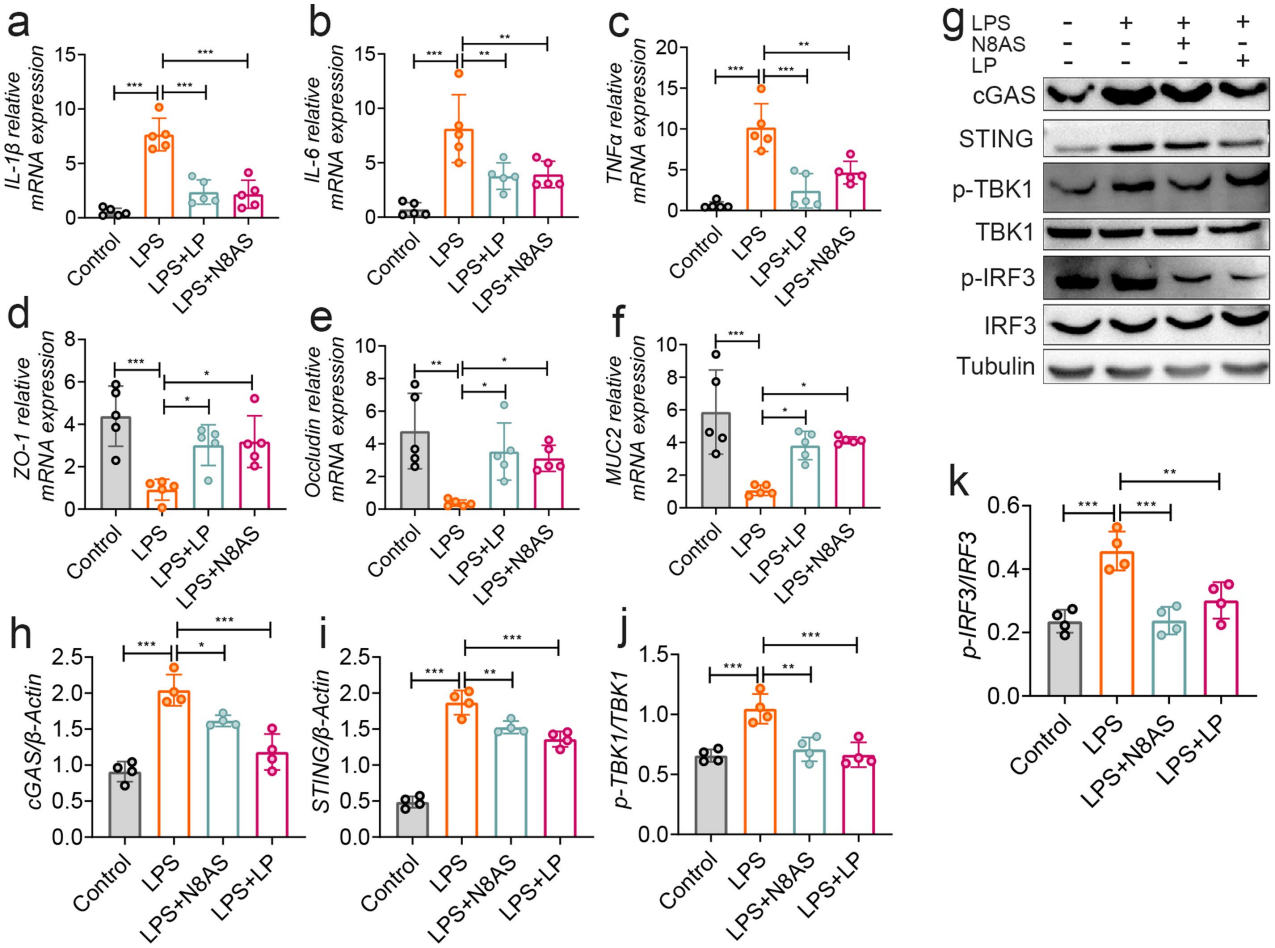
*In vitro* verification, lily polysaccharide can inhibit the cGAS-STING pathway and play an anti-inflammatory role. **(a)** *IL-1β* mRNA expression. **(b)** *TNF-α* mRNA expression level. **(c)** *IL-6* mRNA expression level. **(d)** *ZO-1* mRNA expression level. **(e)** *occludin* mRNA expression level. **(f)** *MUC2* mRNA expression level. **(g–k)** The protein expression levels of cGAS, STING, p-TBK, p-IRF3 were assessed by western blot analysis. Data are presented as the mean ± SEM. Statistically significant differences are indicated; *p < 0.05, **p < 0.01, *** *p* < 0.001.

Based on the *in vivo* findings, we further investigated the molecular mechanisms underlying the anti-colitic effects of LP and N8AS using an LPS-induced *in vitro* inflammation model. Experiments were conducted in Caco-2 intestinal epithelial cells and RAW264.7 macrophages. In LPS-stimulated RAW264.7 macrophages, treatment with LP or N8AS significantly reduced the production of pro-inflammatory cytokines IL-1β, IL-6, and TNF-*α* (Figures 8a–c), confirming their potent anti-inflammatory activity. In Caco-2 cells, both LP and exogenous N8AS markedly upregulated the expression of key epithelial barrier-related genes, including *Occludin*, *ZO-1*, and *MUC2* (Figures 8d–f), suggesting their role in promoting barrier restoration. To elucidate the underlying mechanisms, we further assessed key proteins in the cGAS–STING signaling pathway (cGAS, STING, p-TBK1, and p-IRF3) in RAW264.7 cells. LPS stimulation robustly activated this pathway, whereas treatment with either LP or N8AS effectively suppressed its activation (Figures 8g–k), indicating that N8AS mediates anti-inflammatory effects, at least in part, through inhibition of the cGAS–STING pathway.

Taken together, these findings demonstrate that LP alleviates inflammation by suppressing cGAS–STING signaling, while N8AS, as a downstream effector induced by LP, can independently inhibit this pathway. Thus, LP and N8AS act synergistically to confer immunomodulatory and barrier-protective effects under inflammatory conditions.

## 4 Discussion

This study investigated the therapeutic effects of LP in a murine model of DSS-induced colitis and demonstrated that LP alleviates intestinal inflammation by modulating the gut microbiota and increasing the levels of N8AS. Furthermore, *in vitro* experiments revealed that both LP and N8AS inhibited activation of the cGAS–STING signaling axis, thereby reducing inflammation through suppression of pro-inflammatory cytokine production.

A hallmark of IBD is the marked elevation of pro-inflammatory mediators within the intestinal tissue (Lu et al., 2024). Such mediators can disrupt the intestinal barrier and alter gut microbiota composition, thereby exacerbating disease progression (Aggeletopoulou et al., 2024; Prame Kumar et al., 2023). In this study, DSS-induced colitis in mice was associated with significant upregulation of IL-6, TNF-α, and IL-1*β*. Consistent with previous reports showing that suppression of pro-inflammatory cytokine expression can effectively mitigate DSS-induced colitis (Liu et al., 2024),we observed that oral administration of LP markedly downregulated these cytokines. Moreover, histological analysis and colon length measurements demonstrated that LP treatment attenuated colonic injury and preserved colon length. Immunostaining of intestinal barrier-related proteins further confirmed that LP upregulated their expression, suggesting improved barrier integrity. Collectively, these findings indicate that LP exerts significant anti-inflammatory effects in the intestine, although the precise mechanisms remain to be fully clarified.

The colon is the most microorganism-rich part of the intestinal tract, and alterations in microbial communities directly affect colonic health (Shealy et al., 2024; Yuan et al., 2024). To further elucidate the mechanism by which LP alleviates intestinal inflammatory responses, changes in the gut microbiota were assessed. LP significantly increased the abundance of *Bacteroidota*, with *Bacteroides_H* and *Dubosiella* showing notable increases at the genus level. *Dubosiella* has been reported to regulate metabolism, enhance intestinal immunity, and promote resistance to inflammatory diseases (Tan et al., 2024; Wang Y. et al., 2024). Similarly, Turicimonas has been implicated in host metabolic regulation, with alterations in its abundance associated with changes in amino acid and short-chain fatty acid metabolism, thereby influencing inflammation and energy balance (Lai et al., 2018). These findings suggest that LP can modulate the intestinal flora. To further confirm this, fecal microbiota from LP-treated mice were transplanted into DSS-induced colitis mice. The results demonstrated a significantly improved therapeutic effect in recipient colitis mice compared with controls, indicating that LP alleviates intestinal inflammation at least in part by modulating the structure of the gut microbiota.

Metabolites are crucial active substances that regulate intestinal health (Archana et al., 2024). Previous studies have demonstrated that the body’s metabolites significantly influence intestinal inflammation (Yu et al., 2024). To further clarify the regulatory effects of LP on intestinal metabolites, metabolomics analysis was performed, identifying a total of 650 differential metabolites, including 65 upregulated and 108 downregulated metabolites. These metabolites were predominantly enriched in arginine biosynthesis, D-amino acid metabolism, β-alanine metabolism, tryptophan metabolism, histidine metabolism, alanine, aspartate and glutamate metabolism, vitamin B6 metabolism, and pantothenate and CoA biosynthesis. Further analysis revealed that LP significantly increased the levels of N8AS. Interestingly, previous studies have shown that N8AS levels are higher in naked mole rats than in mice, and that N8AS regulates ischemic cardiomyocyte apoptosis and the resulting cardiac dysfunction (Viltard et al., 2019; Yoshimoto et al., 2021). Moreover, elevated N8AS levels have been observed in colonic epithelial cells of both IBD patients and DSS-induced colitis mice, where they correlate with the severity of mucosal inflammation (Weiss et al., 2004). Since gut microbiota are known to play an important role in polyamine metabolism, it is reasonable to speculate that LP may indirectly elevate N8AS levels by modulating microbial composition and metabolic activity, thereby promoting polyamine biosynthesis and acetylation-related metabolic pathways (Tofalo et al., 2019).

To further verify the role of N8AS in intestinal inflammation, we assessed its effects in a murine colitis model. Oral administration of N8AS significantly alleviated intestinal inflammation in DSS-treated mice. However, the precise mechanism by which N8AS alleviates colitis remains unclear. We therefore examined the cGAS–STING signaling pathway, a key innate immune pathway involved in host defense, immune surveillance, and the pathogenesis of many diseases. Interestingly, the cGAS–STING pathway was significantly inhibited in the colonic tissues of N8AS-treated mice. Given the close relationship between colitis and host immune function, these findings suggest that N8AS may exert protective effects against colitis at least in part by inhibiting the cGAS–STING signaling pathway.

To further confirm the regulatory effects of N8AS and LP on the cGAS–STING pathway, *in vitro* models were established using LPS and colitis microbiota supernatant. The results demonstrated that both LP and N8AS significantly inhibited the expression of IL-1β, TNF-*α*, and IL-6 in RAW264.7 cells. We then examined the expression of proteins related to the cGAS–STING signaling pathway. The findings showed that both LP and N8AS markedly suppressed the expression of cGAS–STING pathway-associated proteins in RAW264.7 cells under both LPS stimulation and colitis microbiota supernatant exposure. These results indicate that LP and N8AS can synergistically inhibit activation of the cGAS–STING pathway, thereby contributing to the remission of colitis.

Studies have shown that activation of the cGAS–STING signaling axis represents a key pathway that exacerbates inflammatory responses and promotes the progression of colitis. Previous research has identified the cGAS–STING axis as a critical regulator of intestinal inflammation (Li C. et al., 2024; Xie et al., 2024). For example, substance P was shown to alleviate DSS-induced colitis by suppressing cGAS-STING activation and downstream ferroptosis in colonic tissue (Lan et al., 2024). Similarly, neutrophil extracellular traps (NETs) were found to trigger colonic barrier dysfunction through cGAS-STING pathway activation, further exacerbating ulcerative colitis (Sun et al., 2024). Overactivation of this pathway leads to the release of numerous pro-inflammatory mediators, which in turn promote cell apoptosis and contribute to the development and persistence of intestinal inflammation.

Previous studies have reported that various natural products, such as polysaccharides, flavonoids, and saponins, can alleviate IBD through modulation of gut microbiota and immune pathways. In contrast, the present study places greater emphasis on the mechanistic actions of LP, particularly highlighting the role of N8AS as a beneficial metabolite in colitis relief. This finding not only deepens the understanding of the anti-colitic mechanisms of LP, but also provides novel insights for the development of IBD therapeutic strategies targeting N8AS.

## 5 Conclusion

In summary, LP can alleviate colitis by regulating Gut microbiota/N8AS/cGAS-STING on one hand. On the other hand, it can synergize with N8AS to inhibit the activation of the cGAS STING signaling axis in cells, thereby alleviating colitis.

## Data Availability

The datasets presented in this study can be found in online repositories. The names of the repository/repositories and accession number(s) can be found at: https://www.ncbi.nlm.nih.gov/, PRJNA1332415; https://www.ebi.ac.uk/metabolights/, MTBLS13064.

## Footnotes

^1^ https://www.genescloud.cn/
