# Supplementary material for: Lily polysaccharides alleviate colitis through the microbiota–N8-acetylspermidine–cGAS–STING signaling axis
Source: Front Microbiol. 2025 Nov 3;16:1686902. doi: 10.3389/fmicb.2025.1686902 (PMC12620830; doi:10.3389/fmicb.2025.1686902)
Supplement: Supplementary file 1 [file Data_Sheet_1.docx]

**Supplementary Materials**

**Figure S1. Phylogenetic tree plot. “B” represents DSS+LP group, “D” represents DSS group.**

**
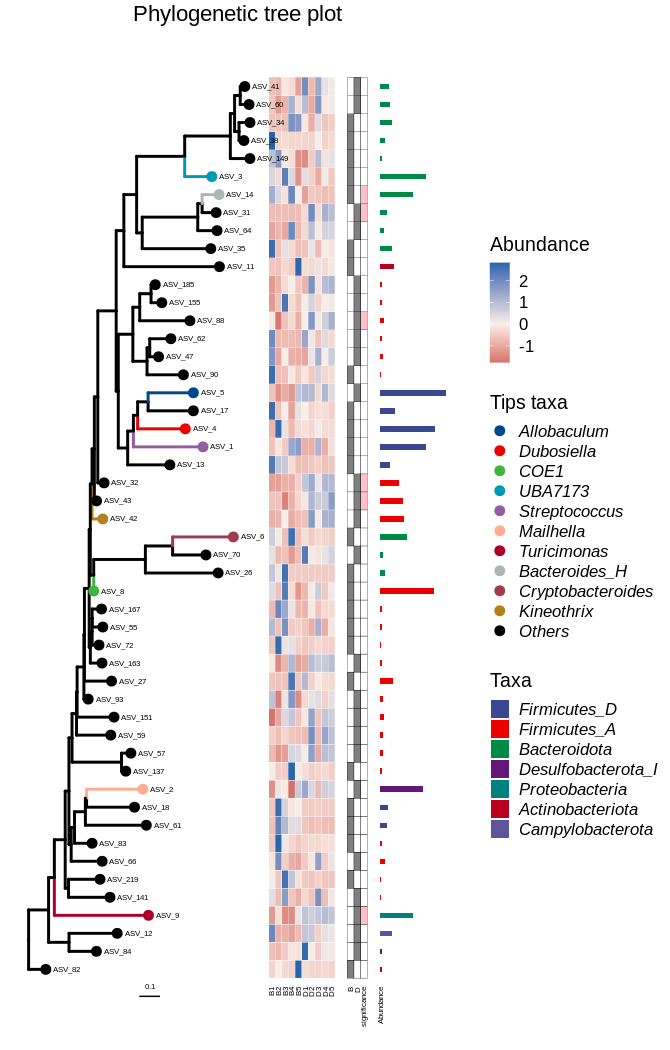
**

**Fig. S2. Differential species abundance histogram. “B” represents DSS+LP group, “D” represents DSS group.**

**
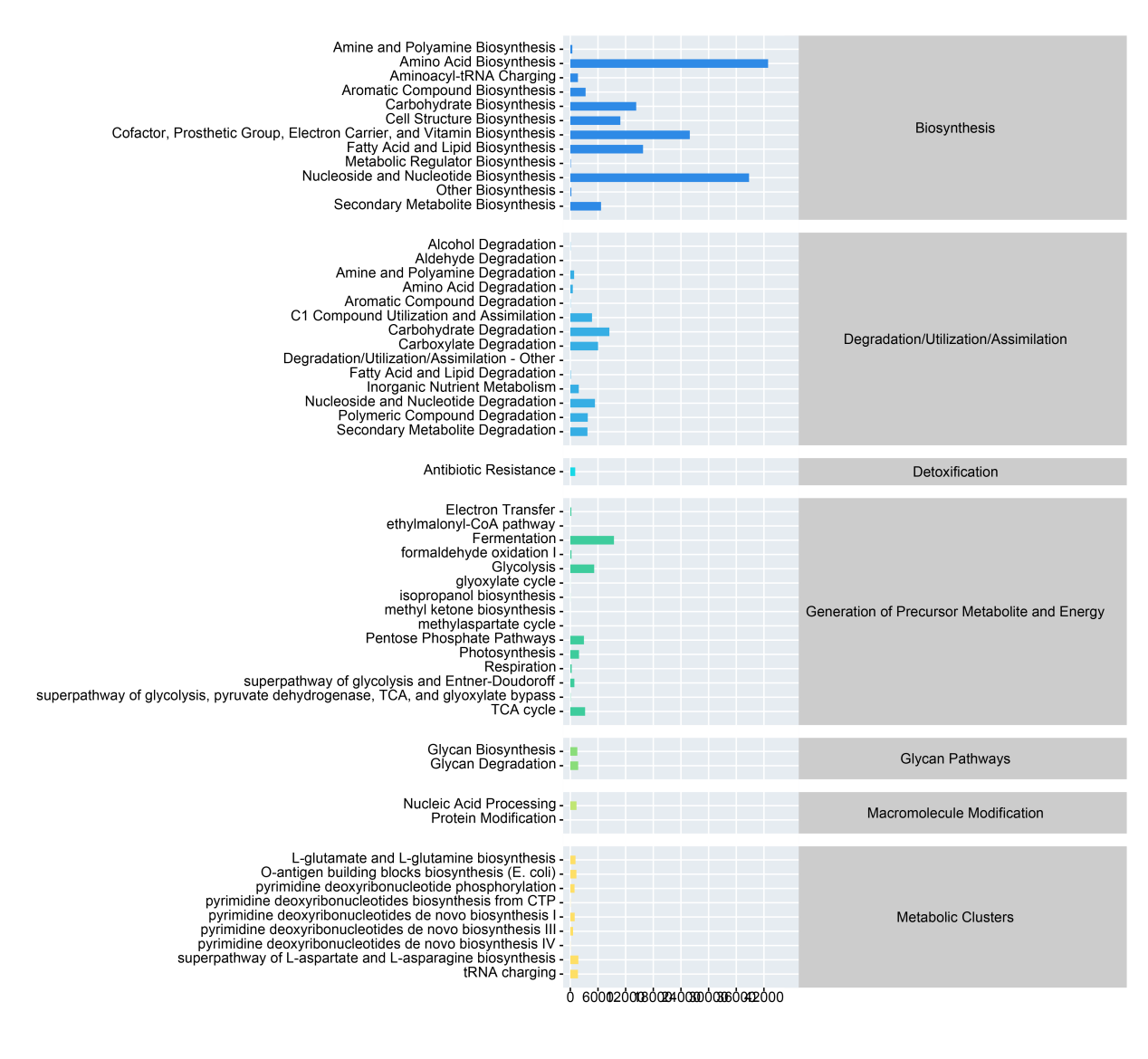
**
